# Life Course Socioeconomic Position and C-Reactive Protein: Mediating Role of Health-Risk Behaviors and Metabolic Alterations. The Brazilian Longitudinal Study of Adult Health (ELSA-Brasil)

**DOI:** 10.1371/journal.pone.0108426

**Published:** 2014-10-13

**Authors:** Lidyane V. Camelo, Luana Giatti, Jorge Alexandre Barbosa Neves, Paulo A. Lotufo, Isabela M. Benseñor, Dóra Chor, Rosane Härter Griep, Maria de Jesus Mendes da Fonseca, Pedro Guatimosim Vidigal, Ichiro Kawachi, Maria Inês Schmidt, Sandhi Maria Barreto

**Affiliations:** 1 Postgraduate Program in Public Health, Faculty of Medicine, Universidade Federal de Minas Gerais, Belo Horizonte, Minas Gerais, Brazil; 2 Department of Social and Behavioral Sciences, Harvard School of Public Health, Boston, Massachusetts, United States of America; 3 School of Nutrition, Universidade Federal de Ouro Preto, Ouro Preto, Minas Gerais, Brazil; 4 Faculty of Philosophy and Human Sciences, Universidade Federal de Minas Gerais, Belo Horizonte, Minas Gerais, Brazil; 5 Center for Clinical and Epidemiologic Research, Hospital Universitário, Universidade de São Paulo, São Paulo, São Paulo, Brazil; 6 Escola Nacional de Saúde Pública, Fundação Oswaldo Cruz, Rio de Janeiro, Rio de Janeiro, Brazil; 7 Laboratory of Health and Environment Education, Fundação Oswaldo Cruz, Rio de Janeiro, Rio de Janeiro, Brazil; 8 Department of Epidemiology and Quantitative Methods in Health, Escola Nacional de Saúde Pública, Fundação Oswaldo Cruz, Rio de Janeiro, Rio de Janeiro, Brazil; 9 Department of Laboratory Medicine, Faculty of Medicine, Universidade Federal de Minas Gerais, Belo Horizonte, Minas Gerais, Brazil; 10 Postgraduate Studies Program in Epidemiology, School of Medicine, Universidade Federal do Rio Grande do Sul, Porto Alegre, Rio Grande do Sul, Brazil; University of Glasgow, United Kingdom

## Abstract

**Background:**

Chronic inflammation has been postulated to be one mediating mechanism explaining the association between low socioeconomic position (SEP) and cardiovascular disease (CVD). We sought to examine the association between life course SEP and C-reactive protein (CRP) levels in adulthood, and to evaluate the extent to which health-risk behaviors and metabolic alterations mediate this association. Additionally, we explored the possible modifying influence of gender.

**Methods and Findings:**

Our analytical sample comprised 13,371 participants from ELSA-Brasil baseline, a multicenter prospective cohort study of civil servants. SEP during childhood, young adulthood, and adulthood were considered. The potential mediators between life course SEP and CRP included clusters of health-risk behaviors (smoking, low leisure time physical activity, excessive alcohol consumption), and metabolic alterations (obesity, hypertension, low HDL, hypertriglyceridemia, and diabetes). Linear regression models were performed and structural equation modeling was used to evaluate mediation. Although lower childhood SEP was associated with higher levels of CRP in adult life, this association was not independent of adulthood SEP. However, CRP increased linearly with increasing number of unfavorable social circumstances during the life course (p trend <0.001). The metabolic alterations were the most important mediator between cumulative SEP and CRP. This mediation path accounted for 49.5% of the total effect of cumulative SEP on CRP among women, but only 20.2% among men. In consequence, the portion of the total effect of cumulative SEP on CRP that was mediated by risk behaviors and metabolic alterations was higher among women (55.4%) than among men (36.8%).

**Conclusions:**

Cumulative SEP across life span was associated with elevated systemic inflammation in adulthood. Although health-risk behaviors and metabolic alterations were important mediators of this association, a sizable fraction of this association was not mediated by these factors, suggesting that other pathways might play a role, especially among men.

## Introduction

The association between lower adulthood socioeconomic position (SEP) and increased risk of cardiovascular disease (CVD) is well-established [1]. Exposure to disadvantaged socioeconomic circumstances during childhood and youth have also been shown to be powerful predictors of CVD [2], indicating that SEP acts across the life course, rather than just in adulthood.

A number of mechanisms have been put forward to account for the association between low life course SEP and cardiovascular risk, including higher prevalence of risk behaviors among disadvantaged individuals, such as smoking, excessive alcohol consumption, and sedentarism [3]. These behaviors may in turn lead to metabolic, endocrine and immune dysregulation, which could promote a pro-inflammatory and pro-thrombotic state [3], [4]. Some evidence also suggests that chronic stress associated with socioeconomic adversity leads to epigenetic modifications affecting the transcription of the glucocorticoid receptor leading to glucocorticoid resistance. This phenotype may deregulate the neuroendocrine feedback governed by the hypothalamic-pituitary-adrenal axis resulting in elevated secretion of cortisol as well as pro-inflammatory cytokines such as interleukin-6 [5]–[9].

Interleukin-6 is one of the most important factor involved in the induction of synthesis of the C-reactive protein (CRP), an acute-phase reactant protein produced mainly by the liver [10]. Although the possible role of CRP as a causal factor for CVD remains debated [11], [12], extensive evidence suggests that CRP serves as a marker of inflammation and their levels predict the incidence of CVD [13], [14]. In high-income countries, the association between low life course SEP and elevated levels of CRP has been extensively investigated [15]–[20]. However, there is a lack of consistency among these studies with regard to the persistence of this association after controlling for the effect of health-related behaviors and metabolic alterations (such as obesity, hypertension, diabetes and dyslipidemia). Most studies found no remaining association between SEP and CVD after considering the effect of these variables, especially of obesity [15], [16], [19]. These findings suggest that health-related behaviors and metabolic alterations fully mediate the relation between life course SEP and chronic inflammation.

There is also uncertainty as to the existence of critical periods, during which SEP would exert an irreversible and independent influence on the development of chronic inflammation, or of sensitive periods, during which SEP would exert a stronger influence on chronic inflammation [21], [22]. Some studies found that exposure to unfavorable social circumstances in childhood was associated with higher CRP levels independently of adulthood SEP [17], [23], [24] and that current SEP was not associated with CRP levels after considering the influence of childhood SEP [17], [23], [24]. Yet other studies found that only adulthood SEP has an influence on CRP levels [16]. Other studies have also suggested a cumulative influence of socioeconomic disadvantage on CRP levels, i.e. the greater the exposure of disadvantage across the life course, the higher the CRP level [16]–[18], [25].

Brazil, like other upper-middle income countries, has faced great economic and demographic changes in recent decades. It has shifted from a predominantly rural to an urban country with a rapidly aging population. Inequality and poverty levels have decreased sharply in recent years due to anti-poverty policies including increases in the minimum wage, cash transfer programmers, and improvements in the public health system [26], [27]. Thus, an important fraction of the population has experienced recent upward socioeconomic mobility. However, the country remains among the highest in the world in terms of income inequality, with a national Gini index of 0.51 in 2012 [28].

The association between SEP and obesity in Brazil differ by gender, and whereas among women there is a clear inverse relation between SEP and obesity, among men SEP is directly or not associated at all with obesity [29], [30]. In addition, the association between CRP and obesity is higher in women in many North American and European studies [31], and the obesity has been shown to be the most important predictor of CRP [32]–[34]. Thus, the association between SEP and CRP might differ among men and women. This gender difference was supported by results from the 1982 Pelotas (Brazil) Birth Cohort Study (mean age = 22.7 years). In this study childhood SEP and CRP were not associated in women, whereas among men there was an association, but in the opposite direction of what has been observed in developed countries: i.e. men reporting higher family income at birth presented higher levels of CRP in adult life independently of current SEP and metabolic alterations [23]. The explanation for this unexpected result remains unclear and further investigation is needed especially in middle aged adults, when SEP is more stable.

Thus, our aim was to evaluate the association of socioeconomic position across the life course with CRP levels in adulthood among middle aged civil servants living in a higher middle income country undergoing rapid transformation. Specifically, our objective was to investigate whether there is a critical period when exposure to lower SEP more strongly influences CRP levels, and/or if there is evidence of a cumulative SEP effect. Additionally, we investigated whether health-risk behaviors and metabolic alterations potentially mediate the association between life course SEP and chronic inflammation, and whether gender modifies this relationship.

## Methods

### Data source and study population

This study used the baseline data from ELSA-Brasil. The design and selection criteria of ELSA-Brasil were described elsewhere [35], [36]. Briefly, 15,105 civil servants, aged between 35 and 74, active or retired, were enrolled from universities and research institutes in six Brazilian states (São Paulo, Minas Gerais, Bahia, Rio Grande do Sul, Rio de Janeiro and Espírito Santo). The baseline examination (2008–2010) included detailed interviews, as well as clinical, laboratory and anthropometric examinations.

### Exclusion Criteria

From the 15,105 participants at baseline, we excluded from this analysis 1263 women who were using hormonal contraceptive therapy or hormonal replacement therapy at the time of the blood draw, as this group has been shown to have elevated CRP levels [37], [38]. In addition, we excluded 108 participants for having missing values for CRP, and 363 for having CRP values below the detection limit (0.175 mg/L). Thus, 1,734 participants were excluded (233 men and 1501 women) and the analysis sample comprised 13,371 (88.5%) participants.

The excluded men were similar to those included with regard maternal education, occupational social class in the first job, current occupational social class, and own education attainment. However, excluded men were more likely to have higher *per capita* household income (p = 0.035). In comparison with the women participants, those excluded presented higher maternal education (p<0.001), higher occupational social class in the first job (p<0.001), higher own education attainment (p<0.001), higher current social class (p<0.001), and higher *per capita* household income (p<0.001).

### Study Variables

#### CRP levels

Serum CRP was obtained from overnight fasting blood and was measured using high-sensitivity assay by immunochemistry - nephelometry - (BN II; Siemens).

#### Life course SEP indicators

Childhood SEP: Maternal education was used as an indicator of childhood SEP, and it was assessed retrospectively by self-report, using years of schooling, based on the question “*What is the educational level of your mother?*.”

Young adulthood SEP: Participants' own education and occupational social class of the first job were used to measure young adulthood SEP. Participants' own education was obtained by self-report, in years of schooling, using the question “*What is your education level?*”. Occupational social class of the first job is a summary measure based on the first job held by the participant, obtained using the open question: *“What was your occupation or activity on your first job?*. It considers the relationship schooling-income by comparing the expected income based on the educational level required by the job and the observed income prevailing in the labor market. These scores were categorized into 7 levels (high-upper, high-low, middle-upper, middle-middle, middle-low, low-high and low-low) [39].

Adulthood SEP: Current occupational social class and *per capita* household income were used to evaluate adulthood SEP. The current occupational social class was obtained using the same approach that was used to obtain the social class of the first job, but using the current occupation, obtained by the open question: “*Please describe the main activities that you develop in your day-to-day work at this institution*”. The net household income was evaluated by self-report using the question: *“During the last month, what was, approximately, your net household income, that is, the sum of incomes, already considering tax discounts, of all the people who regularly contribute with house expenses?”* and the *per capita* household income was obtained dividing this amount by the total number of people living in the household.

Cumulative SEP score: To indicate the accumulation of risk during the life course, a cumulative SEP score was generated and ranged between zero to nine (higher values reflecting worse life course SEP and higher risk), and including maternal education (≥11 years of study = 0; 8–10 years of study = 1; 1–7 years of study = 2; 0 years of study = 3), participant's own education (≥15 years of study = 0; 11–14 years of study = 1; 8–10 years of study = 2; 0–7 years of study = 3), and per capita household income (4th quartile = 0; 3rd quartile = 1; 2nd quartile = 2; 1st quartile = 3).

#### Potential mediators

Health-risk behaviors: current cigarette smoking was determined by self-report if the participants declared having smoked at least 100 cigarettes in their lifetime and still smoked at the time of the research. Physical activity was measured using the International Physical Activity Questionnaire (IPAQ) – Short Form, and low leisure time of physical activity was defined according to the IPAC Guidelines for Data Processing and Analysis [40], as participants who did not meet any of the following three criteria: 3 or more days of vigorous activity during the last week, consisting of at least 20 minutes per day; or 5 or more days of moderate-intensity activity and/or walking during the last week, consisting of at least 30 minutes per day; or 5 or more days of any combination of walking, moderate or vigorous-intensity activities during the last week, achieving a minimum of at least 600 Metabolic Equivalent of Task (MET)-minutes per week [40]. The alcohol consumption was evaluated by self-report of usual type, frequency of intake, and drinking patterns. All the information obtained was summarized in quantity of grams of alcohol drank per week. Excessive alcohol consumption was defined as consuming ≥210 g of alcohol per week among men, and ≥140 g per week among women. To indicate the cluster of these health-risk behaviors, we created a score that ranged from 0 (absence of health-risk behavior) to 3 (presence of all three health-risk behaviors).

Metabolic alterations: anthropometric measurements of weight, height and waist circumference were used to define “obesity/abdominal obesity” as participants who presented body mass index ≥30 kg/m^2^ and/or waist circumference ≥88 cm for women and ≥102 for men. Hypertension was defined as systolic blood pressure ≥140 mmHg or diastolic blood pressure ≥90 mmHg or verified treatment with anti-hypertensive medication. Low HDL cholesterol was defined as HDL <40 mg/dL for men and <50 mg/dL for women. Hypertriglyceridemia was defined as ≥150 mg/dL. Diabetes was defined as a self-report of a previous diagnosis of diabetes or the use of medication for diabetes or fasting glucose ≥126 mg/dL or glucose tolerance test ≥200 mg/dL or glycated hemoglobin ≥6.5%. To indicate the cluster of these metabolic alterations, we generated a score that ranged from 0 (absence of metabolic alterations) to 5 (presence of all five metabolic alterations).

### Data Analyses

We generated descriptive characteristics of the analytic sample. Categorical variables were summarized as frequencies and continuous variables were summarized as means and standard deviation (SD) or median and interquartile range (IQR). All analyses were conducted separately for men and women to explore the possible modifying influence of gender.

The prevalence of each health-risk behavior and metabolic alteration was described according to the cumulative SEP score. We compared the median CRP levels according to the presence or absence of each of the health-risk behaviors and metabolic abnormality. The statistical significance of the differences between the median values in those groups was evaluated using the Wilcoxon rank-sum test, since the levels of CRP were left-skewed.

CRP was natural log-transformed due to non-normality. We estimated the age-adjusted geometric means of CRP for each SEP indicator by exponentiating the parameter estimates from linear regression models on natural log-transformed CRP (back- transformed). We also examined geometric means of CRP adjusted for age and all SEP indicators simultaneously. The adjustment for age was necessary, since CRP increases with age [10], and socioeconomic position also differed according to age. For example, educational attainment varies by births cohort, and older people tend to have lower education than the young people in the ELSA-Brasil cohort.

To estimate the age-adjusted geometric means of CRP, the maternal education was grouped in four categories (≥11, 8–10, 1–7, 0 years of study), as well as the participants' own education attainment (≥15, 11–14, 8–10, 0–7 years of study). The occupational social class of the first job and the current occupational social class were summarized in three categories (high, middle, low), and the *per capita* household income was categorized into quartiles. However, to test the linear trends of these CRP means by SEP we entered the SEP indicators as continuous variable in these models. The normality of residuals and homoscedasticity were tested graphically and violation was not found. The multicollinearity between the explanatory variables was assessed by the variance inflation factor (VIF) and all VIF values were far below 10, the critical value for a serious problem of multicollinearity [41].

#### Mediation Analyses

We used structural equation modeling to test the hypothesis that the association between cumulative SEP and CRP is partly mediated by health-risk behaviors and metabolic alterations.

**Figure 1:**
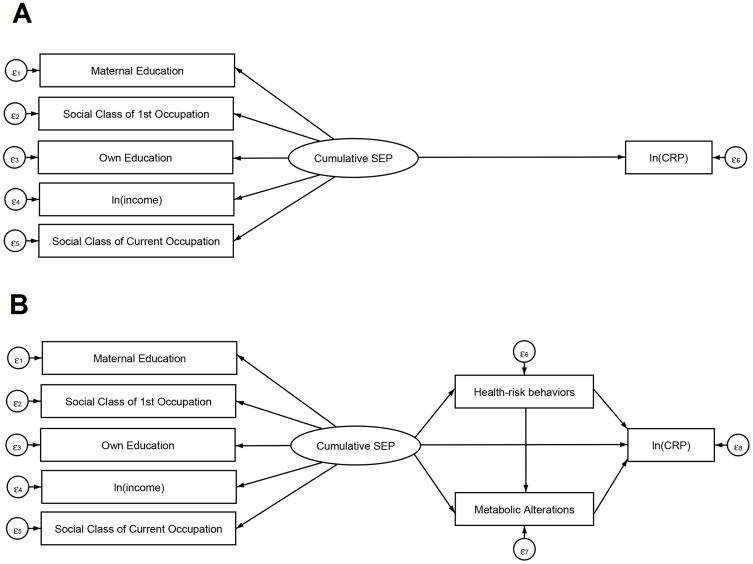
Illustration of proposed multiple mediation of the association between life course SEP and CRP. (A) Total effect of life course SEP on CRP. (B) Hypothesized indirect effect of SEP on CRP through mediators and direct effect. Brazilian Longitudinal Study of Adult Health (ELSA-Brasil), 2008–2010.

A latent variable was created in the measurement model to represent the cumulative SEP and included maternal education, participant's own education, occupational social class of the first job, current occupational social class and per capita household income. All SEP indicators were included in the measurement model as continuous variables, and the *per capita* household income was natural log-transformed due to non-normality. The scores created to access the clustering of health-risk behaviors and metabolic alterations were used in the structural equation models. Figure 1 shows details of the model that was tested. Figure 1A shows the total effect of cumulative SEP on CRP. Figure 1B shows that the estimate of the total effect was disaggregated into three indirect effects, which represent the effects mediated by health-risk behaviors and metabolic alterations (Cumulative SEP = >Risk Behavior = >ln(CRP); Cumulative SEP = >Metabolic alterations = >ln(CRP); Cumulative SEP = >Risk Behavior = >Metabolic alterations = >ln(CRP)), and the remaining direct effect of cumulative SEP on CRP that is independent of these mediators. Despite the a priori importance of age as pointed out above, the age standardized coefficient was not statistically significant in the mediation models. For this reason, we did not include age in the mediation analysis, because this inclusion did not materially alter the other estimates, but affected the model adjustment because of the existence of a non-significant variable in the model.

The maximum likelihood procedure was used to estimate the structural equation model parameters. Standardized coefficients with 95%CI, and tests of significance for standardized coefficients were reported. The absence of overlap in the 95%CI for each standardized coefficient was interpreted as evidence of a significant gender difference in a given path. Overall model fit was assessed using the Comparative Fit Index (CFI), the Root Mean Square Error of Approximation (RMSEA), and the standardized root mean squared residual (SRMR). For goodness of fit, we followed the recommendation of a CFI ≥0.95, RMSEA ≤0.05, and the SRMR ≤0.08 [42].

All analyses were conducted using the software Stata 12.0 (Stata Corporation, College Station, United States).

### Sensitivity Analyses

In epidemiologic research of chronic inflammation, CRP above 10 mg/L has been considered as acute inflammation and excluded from studies. Nevertheless, recent studies suggest that, especially in obese women, CRP above 10 mg/L can occur due to chronic inflammation [38]. Thus, we showed the results including participants with CRP above 10, but all the analyses were repeated excluding these individuals to verify for possible changes in the results.

### Ethics

ELSA-Brasil research protocol was approved by the Research Ethics Committee of Universidade de São Paulo (USP), Research Ethics Committee of Universidade Federal de Minas Gerais (UFMG), Research Ethics Committee of Fundação Oswaldo Cruz (FIOCRUZ), Research Ethics Committee of Universidade Federal do Espírito Santo (UFES), Research Ethics Committee of Universidade Federal da Bahia (UFBA), Research Ethics Committee of Universidade Federal do Rio Grande do Sul (UFRGS) and also by the National Research Ethics Committee (CONEP). Informed consent was signed by all participants.

## Results

The baseline characteristics of the 13,371 participants (6,654 men and 6,717 women) from the ELSA-Brasil, stratified by sex, are presented in Table 1. The mean age was 52 years (range 35–74 years) and 39.9%of the participants were between 45 and 54 years old. Overall, more than 50% of the participants' mothers had less than eight years of schooling, but their own education attainment was high and over 50% of them had ≥15 years of schooling. On average, the participants had 17 years when they started working. The majority had low social class in their first job, more so among men. On the other hand, about one third of male and one quarter of female social class of current occupational were classified as high. Men reported higher prevalence of smoking and excessive alcohol consumption; while women reported higher prevalence of low leisure time physical activity. The clustering of two or three risk behaviors was substantially more frequent among men than women. The prevalence of obesity/abdominal obesity and low HDL were higher in women than in men. Nevertheless, hypertension and diabetes were more common among men and the prevalence of hypertriglyceridemia was twice that of women.

**Table 1:** Descriptive characteristics of the analytical sample from the Brazilian Longitudinal Study of Adult Health (ELSA-Brasil), N (%) or mean (SD), 2008–2010 (N = 13,371)^1^.

Characteristics	Overall N = 13,371	Men N = 6,654	Women N = 6,717
*Age (years), (N = 13,371), %*			
35–44	2,829 (21.16)	1,490 (22.39)	1,339 (19.93)
45–54	5,338 (39.92)	2,606 (39.16)	2,732 (40.67)
55–64	3,753 (28.07)	1,806 (27.14)	1,947 (28.99)
65–74	1,451 (10.85)	752 (11.30)	699 (10.41)
*Maternal education(years of study),(N = 13,029), %*			
≥11	3,027 (23.23)	1,624 (25.16)	1,403 (21.34)
8–10	2,531 (19.43)	1,248 (19.33)	1,283 (19.52)
1–7	5,567 (42.73)	2,637 (40.85)	2,930 (44.57)
0	1,904 (14.61)	946 (14.66)	958 (14.57)
*Participants' own education (years of study), (N = 13,371), %*			
≥15	6,850 (51.23)	3,359 (50.48)	3,491 (51.97)
11–14	4,695 (35.11)	2,193 (32.96)	2,502 (37.25)
8–10	967 (7.23)	554 (8.33)	413 (6.15)
0–7	859 (6.42)	548 (8.24)	311 (4.63)
*Age at first job, (years), (N = 13,343), mean (SD)*	17.15 (4.87)	16.16 (4.65)	18.13 (4.88)
*Social class of first occupation, (N = 10,710), %*			
High	627 (5.85)	343 (6.09)	284 (5.59)
Middle	3,471 (32.41)	1,474 (26.19)	1,997 (39.30)
Low	6,612 (61.74)	3,812 (67.72)	2,800 (55.11)
*Social class of current occupation, (N = 12,605), %*			
High	3,909 (31.01)	2,291 (35.85)	1,618 (26.04)
Middle	5,397 (42.82)	2,235 (34.97)	3,162 (50.89)
Low	3,299 (26.17)	1,865 (29.18)	1,434 (23.08)
*Per capita household income in U.S. dollars, (N = 13,307), mean (SD)*	896.69 (747.54)	869.51 (708.65)	923.61 (783.31)
*Cumulative SEP score* 2 *(N = 12,971), %*			
0–1 (lowest risk)	2,755 (21.24)	1,473 (22.92)	1,282 (19.59)
2–3	3,739 (28.83)	1,811 (28.18)	1,928 (29.46)
4–5	3,238 (24.96)	1,440 (22.41)	1,798 (27.47)
6–7	2,467 (19.02)	1,223 (19.03)	1,244 (19.01)
8–9 (highest risk)	772 (5.95)	479 (7.45)	293 (4.48)
*Smoking (N = 13,370), %*	1,810 (13.54)	957 (14.38)	853 (12.70)
*Low leisure time physical activity (N = 13,169), %*	10,196 (77.42)	4,835 (73.74)	5,361 (81.08)
*Excessive Alcohol Consumption(N = 13,346), %*	1,051 (7,88)	815 (12.26)	236 (3.52)
*Clustering of unhealthy behaviours* 3 *(N = 13,148), %*			
0	2,520 (19.17)	1,416 (21.62)	1,104 (16.73)
1	8,526 (64.85)	3,892 (59.41)	4,634 (70.24)
2	1,835 (13.96)	1,045 (15.95)	790 (11.98)
3	267 (2.03)	198 (3.02)	69 (1.05)
*Obesity/Abdominal Obesity (N = 13,367), %*	5,158 (38,59)	1,964 (29.53)	3,194 (47.56)
*Hypertension (N = 13,358), %*	4,959 (37,12)	2,697 (40.57)	2,262 (33.71)
*Low HDL (N = 13,367), %*	2,390 (17.88)	992 (14.92)	1,398 (20.82)
*Hypertriglyceridemia (N = 13,366), %*	4,372 (32.71)	2,764 (41.56)	1,608 (23.94)
*Diabetes (N = 13,370),%*	2,758 (20.63)	1,567 (23.55)	1,191 (17.73)
*Clustering of metabolic alterations* 4 *(N = 13,347), %*			
0	3,760 (28.17)	1,733 (26.11)	2,027 (30.21)
1	3,739 (28.01)	1,922 (28.95)	1,817 (27.08)
2	2,964 (22.21)	1,508 (22.72)	1,456 (21.70)
3	1,808 (13.55)	951 (14.33)	857 (12.77)
4	867 (6.50)	448 (6.75)	419 (6.25)
5	209 (1.57)	76 (1.14)	133 (1.98)

1 Differences in total N for each variable are due to missing values.

2 The cumulative SEP score ranged between zero to nine, with higher values reflecting worse life course SEP.

3 It includes current cigarette smoking, low leisure time physical activity, excessive alcohol consumption.

4 It includes obesity/abdominal obesity, hypertension, low hdl, hypertriglyceridemia, diabetes.

The prevalence of health-risk behaviors and metabolic alterations rose with increasing exposure to social adversities across the life course. The only exceptions were obesity in men, which was not associated with cumulative SEP score, and excessive alcohol consumption in women, which was directly associated with life course SEP (Table 2).

**Table 2:** Prevalence of health-risk behavior and metabolic alteration according to cumulative socioeconomic position (SEP) score (higher values reflecting worse life course SEP) among men and women.

Characteristic	0–1	2–3	4–5	6–7	8–9	p for trend
***Men***						
Cigarette smoking	10.52	11.76	15.76	16.68	23.01	<0.001
Low leisure time physical activity	65.53	68.29	76.05	82.11	85.53	<0.001
Excessive alcohol consumption	11.41	11.49	12.72	14.06	13.15	0.026
Obesity/abdominal obesity	31.39	29.93	27.71	28.64	29.65	0.109
Hypertension	37.14	37.56	38.71	43.99	54.70	<0.001
Low HDL cholesterol	12.22	14.41	15.71	17.01	16.98	<0.001
Hypertriglyceridemia	35.78	41.96	43.71	44.56	42.26	<0.001
Diabetes	18.40	19.93	23.28	27.56	38.62	<0.001
***Women***						
Cigarette smoking	11.00	10.27	12.96	15.51	18.77	<0.001
Low leisure time physical activity	72.81	76.78	83.86	89.81	90.94	<0.001
Excessive alcohol consumption	5.15	3.79	3.06	2.25	2.06	<0.001
Obesity/abdominal obesity	40.80	41.65	50.36	55.14	64.51	<0.001
Hypertension	25.25	29.51	34.20	41.00	57.34	<0.001
Low HDL cholesterol	14.66	17.96	24.03	25.16	27.30	<0.001
Hypertriglyceridemia	19.97	22.21	24.92	26.77	34.81	<0.001
Diabetes	11.86	15.35	17.35	23.23	35.49	<0.001

Brazilian Longitudinal Study of Adult Health (ELSA-Brasil), 2008–2010.

The distribution of CRP levels was skewed to lower levels, in men and women. The median (IQR) of CRP levels were 1.35 mg/L (0.71–2.81) and 1.68 (0.82–3.80) mg/L among men and women, respectively. With the exception of excessive alcohol consumption in women, all health-risk behaviors and metabolic alterations were associated with higher levels of CRP (Table 3). It was also notable that CRP levels were more strongly associated with metabolic alterations in women (Table 3).

**Table 3:** Median CRP Levels (interquartile range) according to the presence or absence of health-risk behavior and metabolic alterations among men and women.

Characteristic	Absent	Present	P-value
***Men***			
Cigarette smoking	1.28 (0.69–2.65)	1.95 (0.99–3.72)	<0.0001
Low leisure time physical activity	1.14 (0.62–2.39)	1.43 (0.75–3.04)	<0.0001
Excessive alcohol consumption	1.31 (0.70–2.71)	1.62 (0.81–3.68)	<0.0001
Obesity/abdominal obesity	1.14 (0.62–2.36)	1.96 (1.06–3.96)	<0.0001
Hypertension	1.16 (0.64–2.42)	1.63 (0.87–3.48)	<0.0001
Low HDL cholesterol	1.31 (0.69–2.68)	1.61 (0.82–3.63)	<0.0001
Hypertriglyceridemia	1.17 (0.63–2.53)	1.60 (0.85–3.21)	<0.0001
Diabetes	1.22 (0.67–2.50)	1.88 (1.00–3.93)	<0.0001
***Women***			
Cigarette smoking	1.65 (0.80–3.76)	1.91 (0.91–4.08)	<0.0040
Low leisure time physical activity	1.29 (0.66–3.03)	1.79 (0.87–4.00)	<0.0001
Excessive alcohol consumption	1.67 (0.82–3.79)	1.90 (0.80–4.30)	0.4331
Obesity/abdominal obesity	1.07 (0.59–2.14)	2.90 (1.41–5.54)	<0.0001
Hypertension	1.39 (0.70–3.16)	2.45 (1.14–4.90)	<0.0001
Low HDL cholesterol	1.51 (0.75–3.45)	2.43 (1.15–5.23)	<0.0001
Hypertriglyceridemia	1.45 (0.73–3.37)	2.59 (1.23–4.91)	<0.0001
Diabetes	1.48 (0.75–3.37)	2.85 (1.31–5.82)	<0.0001

Brazilian Longitudinal Study of Adult Health (ELSA-Brasil), 2008–2010.

**Figure 2:**
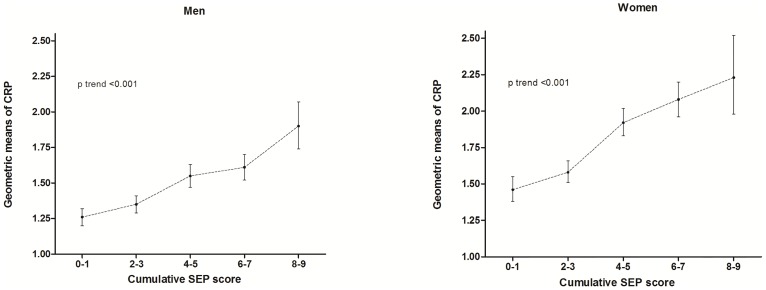
Age adjusted geometric means (95% confidence interval) of C-reactive protein among men and women by the cumulative SEP score that ranged between zero to nine, with higher values reflecting worse life course SEP. Brazilian Longitudinal Study of Adult Health (ELSA-Brasil), 2008–2010.

Age-adjusted geometric means of CRP in adulthood increases with increasing socioeconomic disadvantages in all the life course periods analyzed (Table 4). However, after simultaneous adjustment for all SEP indicators, childhood SEP did not remain statistically associated to CRP in any gender. However, participants' own education, among men, and social class of current occupational and *per capita* household income, among women, remained statistically significantly associated with CRP levels in adulthood (Table 4). In men and women there were cumulative effects of exposure to adverse socioeconomic position across the life span and CRP levels increased linearly with increasing numbers of exposure to unfavorable social contexts over the life course (p for linear trend <0.001) (Figure 2).

**Table 4:** Adjusted geometric means (95% confidence interval) for levels of CRP by SEP indicators throughout the life course.

Indicators	Model adjustment			
	Age		Age and all SEP indicators simultaneously adjusted	
	Men	Women	Men	Women
	Geometric mean (95% CI)	Geometric mean(95% CI)	Geometric mean (95% CI)	Geometric Mean (95% CI)
***Childhood SEP***				
*Maternal education(years of study)*				
≥11	1.31 (1.25–1.37)	1.51 (1.43–1.60)	1.48 (1.39–1.57)	1.72 (1.60–1.86)
8–10	1.49 (1.41–1.57)	1.81 (1.71–1.92)	1.58 (1.48–1.68)	1.84 (1.72–1.97)
1–7	1.47 (1.41–1.53)	1.80 (1.73–1.87)	1.45 (1.39–1.51)	1.77 (1.69–1.85)
0	1.63 (1.53–1.73)	1.95 (1.82–2.09)	1.43 (1.33–1.54)	1.75 (1.61–1.90)
p value for trend	<0.001	<0.001	p = 0.279	p = 0.731
***Young adult SEP***				
*Own education (years of study)*				
≥15	1.29 (1.25–1.34)	1.56 (1.51–1.61)	1.33 (1.25–1.40)	1.71 (1.62–1.80)
11–14	1.59 (1.53–1.66)	1.95 (1.87–2.03)	1.55 (1.47–1.63)	1.79 (1.69–1.89)
8–10	1.68 (1.54–1.82)	2.31 (2.09–2.56)	1.68 (1.52–1.86)	2.13 (1.86–2.44)
0–7	1.87 (1.72–2.03)	2.26 (2.01–2.54)	1.92 (1.72–2.14)	1.92 (1.63–2.27)
p value for trend	<0.001	<0.001	<0.001	p = 0.069
*Social class of first occupation*				
High	1.37 (1.23–1.52)	1.35 (1.19–1.52)	1.60 (1.43–1.79)	1.67 (1.47–1.91)
Middle	1.36 (1.29–1.43)	1.68 (1.60–1.76)	1.44 (1.36–1.52)	1.77 (1.68–1.86)
Low	1.55 (1.50–1.59)	1.90 (1.83–1.98)	1.48 (1.43–1.53)	1.78 (1.71–1.86)
p value for trend	<0.001	<0.001	p = 0.850	p = 0.325
***Adulthood SEP***				
*Social class of current occupation*				
High	1.24 (1.19–1.56)	1.39 (1.32–1.46)	1.37 (1.29–1.47)	1.55 (1.44–1.67)
Middle	1.56 (1.50–1.63)	1.87 (1.81–1.94)	1.58 (1.50–1.65)	1.86 (1.78–1.94)
Low	1.63 (1.56–1.71)	2.05 (1.94–2.17)	1.48 (1.38–1.58)	1.83 (1.70–1.97)
p value for trend	<0.001	<0.001	p = 0.146	p = 0.005
*Per capita household income*				
4^th^ quartile (highest)	1.28 (1.22–1.35)	1.49 (1.42–1.58)	1.43 (1.34–1.53)	1.63 (1.52–1.75)
3^rd^ quartile	1.39 (1.33–1.46)	1.61 (1.53–1.69)	1.52 (1.44–1.61)	1.65 (1.55–1.76)
2^nd^ quartile	1.51 (1.44–1.59)	1.90 (1.80–2.00)	1.45 (1.37–1.53)	1.86 (1.75–1.99)
1^st^quartile (lowest)	1.66 (1.59–1.74)	2.10 (2.00–2.21)	1.50 (1.41–1.59)	1.93 (1.81–2.06)
p value for trend	<0.001	<0.001	p = 0.270	<0.001

Brazilian Longitudinal Study of Adult Health (ELSA-Brasil), 2008–2010.

Table 5 shows the results of the structural equation models. The factor loadings from the measurement model suggest that each of the individual SEP indicators load highly on the cumulative SEP factor measure. There was a significant total effect between cumulative SEP and ln(CRP), showing that each SD increase in cumulative SEP was associated with a 0.134 SD decrease in ln(CRP), among men, and 0.155 SD, among women. The three indirect paths linking cumulative SEP and ln(CRP) were also statistically significant, and the most important indirect path for both men and women was “Cumulative SEP = >Metabolic alterations = >ln(CRP)”. This indirect path was stronger among women than among men, since it accounted for 49.5% of the total effect of cumulative SEP on ln(CRP), among women, and only 20.2% among men. In consequence, the fraction of the total effect of cumulative SEP on ln(CRP), mediated by health-risk behaviors and metabolic alterations was statistically higher in women (55.4%) than in men (36.8%). The direct effect of cumulative SEP on ln(CRP) was high, especially among men, since it accounted for 63.2% and 44.6% of the total effect of SEP on ln(CRP) among men and women, respectively.

**Table 5:** Parameters estimates from the structural equation model of cumulative SEP on CRP levels in adulthood, according to gender.

	Parameter estimates	
	Men N = 5,128^1^	Women N = 4,534^2^
**Measurementmodel, standardized coefficients(95%CI)** 3		
Cumulative SEP→Maternal education	0.525 (0.504; 0.547)***	0.509 (0.484; 0.534)***
Cumulative SEP→Social class of first occupation	0.553 (0.532; 0.574)***	0.575 (0.552; 0.598)***
Cumulative SEP→Own education	0.858 (0.847; 0.869)***	0.844 (0.831; 0.858)***
Cumulative SEP→ln (income)	0.715 (0.699; 0.730)***	0.634 (0.613; 0.654)***
Cumulative SEP→Social class of current occupation	0.866 (0.855; 0.876)***	0.838 (0.824; 0.851)***
**Strutural Model, standardized coefficients (95%CI)** 3		
*Total Effect*		
Cumulative SEP→ln(CRP)	−0.134 (−0.163; −0.106)***	−0.155 (−0.186; −0.125)***
*Direct Effects*		
Cumulative SEP→ln(CRP)	−0.085 (−0.113; −0.056)***	−0.069 (−0.099; −0.039)***
Risk Behavior→ln(CRP)	0.088 (0.060; 0.115)***	0.043 (0.016; 0.070)***
Metabolic Alterations→ln(CRP)	0.256 (0.230; 0.282)***	0.378 (0.353; 0.404)***
*Indirect Effects*		
Cumulative SEP→Risk Behavior→ln(CRP)	−0.018 (−0.024; −0.012)***	−0.007 (−0.011; −0.002)**
Cumulative SEP→Metabolic Alterations→ln(CRP)	−0.027 (−0.035; −0.019)***	−0.077 (−0.090; −0.064)***
Cumulative SEP→Risk Behavior→Metabolic Alterations→ln(CRP)	−0.004 (−0.006; −0.003)***	−0.002 (−0.004; −0.001)*
Total indirect effects: Cumulative SEP→ln(CRP)	−0.049 (−0.059; −0.040)***	−0.086 (−0.099; −0.073)***
***Log CRP R^2^***	0.095	0.164
**Proportion of the effect of Cumulative SEP on ln(CRP) that was:**		
Mediated by Risk Behavior	13.43%	4.44%
Mediated by Metabolic Alterations	20.16%	49.51%
Mediated by Risk Behavior and Metabolic Alteration simultaneously	3.22%	1.47%
Total indirect effect	36.81%	55.43%
Direct effect	63.19%	44.57%
**Model fit** 4		
CFI	0.981	0.989
RMSEA	0.049	0.035
SRMR	0.020	0.015

Brazilian Longitudinal Study of Adult Health (ELSA-Brasil), 2008–2010.

1 Of the 6,654 men participants, 5128 (77.1%) had complete data available on all covariates used in the structural equation model.

2 Of the 6,717 women participants, 4534 (67.5%) had complete data available on all covariates used in the structural equation model

3 The significance levels shown here are for the standardized solution (*p<0.05, **p<0.01, ***p<0.001). The absence of overlap in the 95%CI was interpreted as evidence of a significant gender difference in a given path (“bolded” in the table).

4 CFI: comparative fit index. RMSEA: root mean square error of approximation. SRMR: standardized root mean squared residual.

### Sensitivity Analyses

Of the total participants considered in this analysis, 2.96% of men and 4.56% of women presented CRP above 10 mg/L. Exclusion of these participants from the analysis did not alter significantly any of the results reported above.

## Discussion

Although lower childhood SEP was associated with higher levels of CRP in adult life, this association was not independent of adulthood SEP. However, childhood SEP seems to play a role in chronic inflammatory states when it was considered together with young adulthood SEP and adulthood SEP, providing support to a model of cumulative effects of exposures to SEP across the life span. The cluster of metabolic alterations was the most important mediator between cumulative SEP and CRP in men and women, but for women this mediation path was stronger than for men. Together, metabolic alterations and health-risk behaviors were important mediators between cumulative SEP and CRP. However, the direct effect of cumulative SEP on CRP was substantial, suggesting that other pathways could play a role, especially among men.

According to the life course approach, a critical period is a time window during which exposures can lead to lasting physiological changes in the organism. In its most stringent form, no excess risk would be observed if exposure occurred in periods outside the window [21], [22]. Our results did not support the notion that childhood is a critical period of exposure to low SEP for three reasons. Firstly, CRP was associated with SEP in all three stages of the life course, not just with childhood SEP. Secondly, after simultaneously controlling for adulthood SEP, no association was found between childhood SEP and CRP, suggesting that the exposure to low SEP in early life could matter because it leads to lower adulthood SEP (the pathways or “chain of risk” hypothesis). Thirdly, we found strong evidence of cumulative risk, indicating that exposure to low SEP at different stages of life accumulate to promote chronic inflammation. All these three aspects of our results are incompatibles with the critical period model, at least in the case of chronic inflammation. However, the results showed an important role of the exposure to low SEP in childhood, since it leads to lasting effect through accumulation of risk. Many previous studies also reported increased of CRP levels with increasing number of adverse SEP conditions throughout life [16]–[19], [25].

Clustering of metabolic alterations and health-risk behaviors were important mediators of the association between cumulative SEP and CRP levels in men and women. Using regression models to measure mediation, it was also found in the *Atherosclerosis Risk in Communities* study (ARIC) that diabetes status, low HDL cholesterol, high BMI, smoking and physical inactivity were important mediators between life course SEP and a score of inflammation which included CRP, von Willebrand fator, fibrinogen, and white blood cell count [20].

In our study, metabolic alterations were the most import mediators of the association between cumulative SEP and CRP. However, while this indirect path accounted for 49.5% of the total effect of cumulative SEP on CRP among women, it accounted for only 20.2% among men. This gender difference may be explained by at least two reasons. Firstly, the association between all metabolic alterations and CRP was stronger in women than in men in the ELSA Brasil, which is consistent with other studies [31], [43], [44]. For example, in the British 1958 Birth Cohort the associations between obesity (body mass index, waist circumference), blood pressure, blood lipids, metabolic syndrome and CRP were twice as strong among women as among men [43]. In addition, recent meta-analysis also showed that in adults the Pearson correlation coefficients between body mass index and ln(CRP) was greater in women than men by 0.24 (CI, 0.09–0.37) on average [31]. Secondly, it is well known that adiposity is a major predictor of CRP [32]–[34], and we found that the prevalence of obesity was higher among women than among men (47.56% *versus* 29.53%). Moreover, obesity was not associated with the accumulation of exposures to low SEP during the life course in men, replicating what is currently found in the Brazilian population as a whole [29]. All these findings may explain the much greater contribution of metabolic disorders as mediating path between Cumulative SEP and CRP in women as compared to men.

The cluster of health-risk behaviors accounted for 13.4% of the total effect of Cumulative SEP on CRP among men and only 4.4% among women. Consistently with other studies [45], [46], we found that excessive alcohol consumption was associated with higher CRP levels and with low life course SEP among men. However, among women, the excessive alcohol consumption was not associated with CRP levels. In addition, excessive alcohol consumption was related with higher life course SEP among women, as it was also reported in the general population of Scotland [45]. Moreover, the prevalence of smoking, as well as the clustering of two or more health-risk behaviors, was higher among men. In sum, these facts could account for the greater role of health-risk behaviors as mediators in the association between cumulative SEP and CRP in men than in women. Different findings were reported by the *National Health and Nutrition Examination Surveys* (NHANES IV) using only measures of adulthood SEP. They found that 55.8% of the association between poverty in adulthood and CRP was mediated by 4 health-related behaviors (smoking, heavy alcohol consumption, poor diet and physical activity) and this indirect effect was higher (87.9%) when education level was used to measure adulthood SEP instead of poverty [47]. In contrast to the NHANES IV analyzes, we have not considered poor diet, and the prevalence of smoking and heavy alcohol consumption among US participants was much higher than that found in ELSA-Brasil. For instance, the prevalence of current smoking ranged from 17.7% to 32.8% among non poor and poor NHANES IV participants while heavy alcohol consumption ranged from 16.8% to 20.4%, respectively [47]. Moreover, they only used measures of adulthood SEP, and although the health-related behaviors are often acquired in adolescence [48], it is known that they are more strongly associated with adulthood SEP than with childhood SEP [49].

An important portion of the association between cumulative SEP and CRP was not mediated by metabolic alterations and health-risk behaviors, suggesting that others pathways could play an important role. Stress was not included in the present analysis and may be a relevant path between SEP and CRP levels. Life course SEP could lead to chronic inflammation by increasing exposure to psychosocial stress factors, such as crowding, growing up in poor neighborhoods, experiences of childhood trauma and abuse, discrimination, job strain, and perceptions of relative deprivation [50]–[52]. Chronic stress activates the hypothalamic-pituitary-adrenal (HPA) axis and the sympathetic nervous systems, resulting in higher secretion of cortisol and catecholamines, setting off a chain of physiological consequences including inflammation, coagulation, and adhesion – the so-called model of allostatic load [53]. For instance, in the Whitehall study, job control explained about 64%, among men, and 51%, among women, of the excess risk for coronary heart disease associated with low versus high occupational group [54]. However, most studies found only a small contribution of stress measures to socioeconomic gradient in health [52].

The epigenetic modification induced by the experience of social adversity is another path that could explain some portion of the direct effect that we found between cumulative SEP and CRP. In general, exposures to environmental stressors tend to lead the epigenomic instability (i.e.: DNA demethylation, histone modification and micro-RNA expression). The organism uses this mechanism to respond to threats and, consequently, to increase the diversity. Nevertheless, this process also has the potential to causes diseases [7]. There is growing evidence that socioeconomic adversity can influence DNA methylation and gene expression, especially in genomic regions regulating the immune function [6], [7]. These studies also indicate that exposures to social adversities across the life course may cause glucocorticoid receptor resistance leading to exaggerated glucocorticoid levels in the organism. Thus, uncontrolled inflammatory responses would be typical characteristic of this phenotype created by epigenetic modification [5]–[9]. Originally it was believed that only exposures to SEP in early life could promote this kind of epigenetic modification; however recent evidence suggests that exposure to SEP in adulthood can also promote epigenomic instability [8], [55]. Nevertheless, the association between epigenetics modifications and SEP tend to be higher when measures of SEP in early life were used [6]–[8].

Some potential limitations of our analysis merit consideration. Firstly, to analyze the role of metabolic alterations and health-risk behaviors as mediators between cumulative SEP and CRP we used clusters of risk factors. In doing so, it became feasible to study the mediation process using conventional structural equation modeling. Our approach has two limitations: 1) the specific effect of each behavior or metabolic alteration could be not accessed; 2) working with clusters we considered that all variables have the same weight, but it is possible that different behaviors or metabolic alterations can have more or less influence on CRP levels. For example, among all metabolic alterations considered, it is known that obesity is a major predictor of CRP [32]–[34]. Thus, it was not possible to detect in this analyses which component is more important to mediate the association between cumulative SEP and CRP. Secondly, we do not have the timing of the onset of health behaviors & metabolic alterations – for example, it's possible that most behaviors began in adolescence, which would point to the need for early intervention. Thirdly, we used only maternal education to measure childhood SEP. Others studies that have used other indicators of SEP in childhood, such as parental occupational status and *in utero* SEP, could provide a better evaluation of the influence of exposure to low childhood SEP in chronic inflammation in adulthood. Fourthly, we used the participant's own education to measure young adulthood SEP, since education is generally complete in late adolescence or in the beginning of adult life [56]. However, the participants from ELSA-Brasil are civil servants from universities and research centers and some positions require post-graduate level education. For this reason, we also used the occupational social class of the first job to capture better the SEP in young adulthood, since the mean age that the participants started to work was 16 years among men and 18 among women. Fifthly, we used only the *per capita* household income to evaluate the participants' financial situation, which did not capture other dimensions such as wealth and assets. Sixthly, the ELSA-Brasil participants are active and retired workers, have retirement plans and average education and income levels higher than that of the general population of Brazil. Thus, people who experienced extreme social difficulties in childhood as well as in adulthood could not be represented in this study. The truncated variability in SEP may have led us to underestimate the magnitude of the associations between life course SEP and CRP levels.

The linear association between number of exposures to low SEP and CRP suggests a cumulative impact of SEP in promoting chronic inflammation. These findings provide one potential biological mechanism to explain the well-established social gradient for CVD. Moreover, it suggests that social interventions in a single time point across the life course may not suffice to deal with the social inequalities in CVD. Our findings extend previous studies by using statistical techniques that allowed us to disentangle the portion of the total effect of cumulative SEP on CRP levels that is mediated by metabolic alterations and health-risk behaviors.
